# *Plasmodium yoelii*-Infected *A. stephensi* Inefficiently Transmit Malaria Compared to Intravenous Route

**DOI:** 10.1371/journal.pone.0008947

**Published:** 2010-01-28

**Authors:** Solomon Conteh, Rana Chattopadhyay, Charles Anderson, Stephen L. Hoffman

**Affiliations:** Sanaria Inc., Rockville, Maryland, United States of America; Sabin Vaccine Institute, United States of America

## Abstract

It was recently reported that when mosquitoes infected with *P. berghei* sporozoites feed on mice, they deposit approximately 100–300 sporozoites in the dermis. When we inoculate *P. yoelii* (Py) sporozoites intravenously (IV) into BALB/c mice, the 50% infectious dose (ID_50_) is often less than 3 sporozoites, indicating that essentially all Py sporozoites in salivary glands are infectious. Thus, it should only take the bite of one infected mosquito to infect 100% of mice. In human subjects, it takes the bite of at least 5 *P. falciparum*-infected mosquitoes to achieve 100% blood stage infection. Exposure to 1–2 infected mosquitoes only leads to blood stage infection in approximately 50% of subjects. If mosquitoes carrying Py sporozoites inoculate 100–300 sporozoites per bite, and 1 to 2 mosquito bites achieve 50% blood stage infection rates, then this would suggest that the majority of sporozoites inoculated by mosquitoes into the dermis are not responsible for a productive infection, or that a significant number of sporozoite-infected mosquitoes do not inoculate any sporozoites. The objective of this study was to determine if this is the case. We therefore studied the infectivity to mice of the bites of 1, 2, 4, or 5–8 Py-infected mosquitoes. The bite of one Py sporozoite-infected mosquito caused blood stage infection in 41.4% (12/29) of mice, two bites infected 66.7% (22/33), four bites infected 75% (18/24), and five to eight bites infected 100% (21/21). These findings demonstrate that inoculation of sporozoites by mosquito bite is much less efficient than IV inoculation of Py sporozoites by needle and syringe. Such data may have implications for determining the best route and dose of administration to humans of our attenuated *P. falciparum* sporozoite vaccine, the scientific basis of which is immunity by bites from irradiated infected mosquitoes, and suggest that the challenge is to develop a method of administration that approximates IV inoculation, not one that mimics mosquito bite.

## Introduction

We are currently developing a metabolically active, non-replicating (attenuated) *Plasmodium falciparum* sporozoite malaria vaccine (PfSPZ Vaccine) [1]. The immunogen in the PfSPZ Vaccine is *P. falciparum* sporozoites isolated from salivary glands of irradiated *Anopheles* mosquitoes. Fifteen of sixteen (94%) human volunteers exposed to the bites of greater than 950 irradiated *Anopheles* species mosquitoes with *P. falciparum* sporozoites in their salivary glands were protected against challenge by the bites of five mosquitoes infected with fully virulent *P. falciparum* sporozoites [2]. Only four of ten volunteers exposed to fewer than 950 bites were protected. Thus, there is a dose response and the numbers of sporozoites inoculated is critical for achieving optimal protection.

The development of the PfSPZ Vaccine requires optimizing the dose and method of administration of the *P. falciparum* sporozoites. In mice, 3 doses of 750 radiation-attenuated *P. yoelii* sporozoites (total number of sporozoites = 2250) administered intravenously (IV) leads to between 90% and 100% protection against virulent challenge [3]. This level of protection can be achieved by intradermal or subcutaneous inoculation of the sporozoites, but more sporozoites are required. Because the development of the PfSPZ Vaccine is based on the results of studies in which humans were immunized by the bite of infected mosquitoes, we reasoned that determining the efficiency of transmission of *P. yoelii* to mice by sporozoite-infected *Anopheles* sp. mosquitoes would serve as a model to help us make predictions on the optimal dose and route of PfSPZ Vaccine in humans. Therefore, in multiple experiments, we assessed the capacity of one to eight *P. yoelii* sporozoite-infected *A. stephensi* to infect mice and determined the 50% infectious dose (ID_50_) of *P. yoelii* sporozoites from these same mosquitoes, when inoculated IV. The results indicate that mosquitoes are far less efficient at transmitting malaria than is an IV injection, and suggest that optimizing the protective efficacy of the PfSPZ Vaccine will require developing a vaccine administration system that is as efficient as IV inoculation.

## Materials and Methods

### Mosquitoes

*Anopheles stephensi* derived from two colonies maintained at Sanaria, and designated as *Anopheles stephensi* (NIJ.SAN01) and *Anopheles stephensi* (SXK.SAN02) were used.

### Parasites

Non lethal *Plasmodium yoelii* 17XNL (clone 1.1) [4] was maintained by alternating passage of the parasites in *A. stephensi* mosquitoes and CD-1 mice (Harlan Laboratories, IN).

### Infection of Mosquitoes

The IACUC committee of the University of Maryland Biotechnology Institute (UMBI), Rockville, Maryland, approved all animal experiments described in the study. UMBI's animal facility was used to house the mice and all experiments were conducted at UMBI. Five day-old heat-selected female *A. stephensi* mosquitoes were fed on 4–6 week-old HSD∶ICR(CD-1®) mice (Harlan Bioproducts, Indianapolis, IN) infected with *P. yoelii* (clone 1.1) [4], and maintained for 14–17 days to allow for sporozoite development.

### Exposure of Naïve BALB/c Mice to Bites of *P. yoelii* Infected Mosquitoes

Six-8 week old BALB/c mice (Harlan Bioproducts, Indianapolis, IN, and NCI, Frederick, MD) were anesthetized by intraperitoneal injections of sodium pentobarbital (Sigma-Aldrich, St. Louis, MO). The ventral surface (primarily abdomen) of each mouse was placed on a screened cardboard container harboring sporozoite-infected female *A. stephensi* mosquitoes, which were allowed to feed for 20 minutes. Mosquitoes that fed on mice were checked for the presence of a blood meal in their abdomen and sporozoites in their salivary glands. In some experiments, the mosquitoes that did not have a blood meal in their abdomens and/or sporozoites in their salivary glands were replaced by another mosquito until the pre-designated number of infected mosquitoes had fed.

### Estimation of Numbers of Sporozoites per Mosquito

#### Individual salivary gland squashes

Mosquitoes that had taken a blood meal were dissected individually. The salivary glands were removed and squashed under a cover slip, and were analyzed under a microscope at 200× magnification. The presence of sporozoites was determined by counting the number of sporozoites (PySPZ) per 5–10 fields and scored according to the following method: 0 = no PySPZ seen in the glands; 1+ = 1–10 PySPZ/field; 2+ = 11–100 PySPZ/field; 3+ = >100 PySPZ/field. It was estimated that, per mosquito, there were approximately 250 fields containing salivary gland material. Thus, a mosquito with a 1+ score would have an estimated minimum of 250 sporozoites/mosquito and a maximum of 2500 sporozoites per mosquito. It should be noted that this scoring system differs from the traditional system [5], in which 1+ = 1–10 PySPZ/mosquito; 2+ = 11–100 PySPZ/mosquito; 3+ = 101–1000 PySPZ/mosquito, and 4+ = >1000 PySPZ/mosquito.

#### Pooled salivary gland preparations

For experiments in which the number of sporozoites per mosquito was determined, salivary glands from 10–350 mosquitoes from the same experiment were pooled and sporozoites were counted in a hemocytometer. To determine the mean number of sporozoites per mosquito, the total number of sporozoites was divided by the number of mosquitoes dissected.

### Assessment of Blood Stage Parasitemia

Infections in mice were determined by examining Giemsa-stained thin blood smears (at 1000× magnification) for the presence of blood stage parasites on days 7 and 14 after exposure to mosquito bites.

### Determination of ID_50_ (50% Infectious Dose)

*P. yoelii* sporozoites were harvested from hand-dissected salivary glands of *A. stephensi* and diluted in E-199 medium without L-glutamine (Quality Biological, Inc. Gaithersburg, MD) with 1% Human Serum Albumin (HSA). Four groups of five 6–8 week old female BALB/c mice were inoculated by the intravenous (IV) route with 24 PySPZ/mouse, 12 PySPZ/mouse, 6 PySPZ/mouse, and 3 PySPZ/mouse respectively. The infections in the mice were determined 14 days later and the ID_50_ was calculated as follows: the % infected mice per group (X/5×100) was plotted against the number of sporozoites inoculated. A non-linear exponential association curve (CurveExpert 1.3) was used to calculate the number of sporozoites required to generate a 50% infection rate (ID_50_). The curve fit included a zero data point (an administration of 0 sporozoites would lead to 0% infection).

## Results

### Efficiency of Transmission of Infection by Mosquito Bite

To determine the number of bites required for the establishment of asexual erythrocytic stage infection in mice, one to eight *A. stephensi* mosquitoes with *P. yoelii* sporozoites in their salivary glands were allowed to feed on the ventral surface (primarily abdomen) of BALB/c mice for twenty minutes. Confirmation that the mosquitoes had taken a blood meal was made by identifying blood in the abdomen of individual mosquitoes dissected immediately after feeding. Confirmation of the presence of sporozoites in the mosquitoes was established by microscopic analysis of salivary gland preparations from individual mosquitoes. In these experiments, only mice that were exposed to blood-fed infected mosquitoes were considered in the analysis. On days 7 and 14 following feeding, blood smears were assessed for asexual erythrocytic-stage parasitemia. The data from 5 separate experiments are shown in **Table 1**. The combined data from the first 4 experiments indicated that 43% (10/23) of mice that received 1 bite developed parasitemia, 63% (17/27) that received 2 bites developed parasitemia, and 64% (9/14) that received 4 bites developed parasitemia. 100% (15/15) of mice that received between 5 and 8 bites developed blood stage parasitemia.

**Table 1:** Infection rates after exposure to the bite of one to eight *P. yoelii*-infected mosquitoes.

experiment #	*A. stephensi* strain	infected/challenged (% infected)			
		1 bite	2 bites	4 bites	5–8 bites
1	NIJ.SAN01	2/9	4/10	N/A	N/A
2	NIJ.SAN01	1/4	7/9	5/7	10/10
3	NIJ.SAN01	6/9	4/6	N/A	N/A
4	NIJ.SAN01	1/1	2/2	4/7	5/5
total (1–4)		10/23 (43%)	17/27 (63%)	9/14 (64%)	15/15 (100%)
5	SXK.SAN02	2/6 (33%)	5/6 (83%)	9/10 (90%)	6/6 (100%)
total (1–5)		12/29 (41.4%)	22/33 (66.7%)	18/24 (75%)	21/21 (100%)

BALB/c mice were exposed to one, two, four, or five to eight bites by *A. stephensi* mosquitoes infected with *P. yoelii* sporozoites. 7 and 14 days following bites, mice were assessed for parasitemia by blood smear. Data from individual mice from five separate experiments (experiments 1–5) are shown. A single strain of *A. stephensi* (NIJ.SAN01) was used in experiments 1–4 and a second strain (SXK.SAN02) in experiment 5.

### Numbers of Sporozoites per Mosquito

Microscopic analyses of salivary gland squashes from each mosquito that had fed on a mouse were performed, and the results demonstrated that the mosquitoes had substantial numbers of sporozoites. The results of the salivary gland scores from the salivary gland squashes from all experiments in which a mouse was exposed to a single mosquito are reported in **Table 2**. Recognizing the sensitivity limitations of this semi-quantitative method, there was no indication that the sporozoite load was different in mosquitoes that transmitted or did not transmit an infection. To further refine our understanding of the numbers of sporozoites per mosquito and to eliminate the possibility that low numbers of sporozoites in the salivary glands of mosquitoes were a contributing factor to the low transmission rate, the mean number of sporozoites per mosquito in each container was determined. Mosquitoes raised in the same containers as the mosquitoes used to bite mice were assessed. Salivary glands were dissected from these mosquitoes and pooled sporozoites were counted. The mean numbers of sporozoites per mosquito from experiments 1 through 5 are shown in **Table 3**. The mean number of sporozoites per mosquito for experiments 1 through 4 was 23,198 and the range from container to container was 16,500 to 27,875. Therefore, insufficient numbers of sporozoites/mosquito was not the reason for the low infection rate. Furthermore, sporozoite burden in the salivary glands appeared not to be a limiting factor for transmission to occur.

**Table 2:** Salivary gland scores of individual mosquitoes in Experiments 1–5.

experiment #	infected/challenged from one mosquito bite	salivary gland scores	
		mice that developed parasitemia	mice that did not develop parasitemia
1	2/9	2, 2	2, 3, 3, 2, 2, 3, 2
2	1/4	3	2, 1, 3
3	6/9	3, 3, 3, 3, 3, 3	3, 3, 1
4	1/1	2	
5	2/6	3, 3	3, 1, 3, 3
geometric mean (range)		2.71 (2–3)	2.21 (1–3)

After feeding, the mosquitoes were dissected to demonstrate that they had taken a blood meal and establish the salivary gland score (1+ to 3+, see Methods). Nine of the 17 mosquitoes that fed on mice that did not develop parasitemia (negatives) had the highest salivary gland score of 3+. The geometric mean salivary gland scores of the mosquitoes were not significantly different between the two groups (p = 0.2856, Wilcoxon Two Sample Test).

**Table 3:** Sporozoite density in infected mosquitoes.

experiment #	number of mosquitoes dissected	total number of Py sporozoites isolated	mean # of Py sporozoites/mosquito
1	350	9,298,450	26,567
2	20	557,500	27,875
3	10	218,500	21,850
4	132	2,178,000	16,500
5	15	1,258,125	83,875

The numbers of sporozoites was determined in salivary glands of mosquitoes from the same container as the mosquitoes used in the experiments. Salivary glands from the indicated number of mosquitoes were pooled, sporozoites were isolated from the salivary glands, the total numbers of sporozoites were determined, and the mean numbers of sporozoites/mosquito were calculated.

### Infection Rates with Mosquitoes Derived from a Second Colony

It was possible that the inefficient transmission observed was due to factors in the mosquitoes that were specific to our mosquito colony. To address this, a separate source of *A. stephensi* was identified, *A. stephensi* eggs were obtained, and mosquitoes were reared in our insectary. The mosquitoes were infected by feeding on parasitemic mice, and the experiment was conducted as above. Infection rates in mice exposed to 2 (5/6) or 4 bites (9/10) were higher than previously observed (**Table 1** **, Experiment 5**), but it still required at least 5 bites to achieve infection of 100% of the mice, and only 33% (2/6) of mice became parasitemic following a single mosquito bite. Mosquitoes in this experiment had an average of 83,875 sporozoites per salivary gland (**Table 3**). Therefore, despite the presence of large numbers of sporozoites in the salivary glands, transmission by a single bite by these mosquitoes reared from eggs from a different colony was also not efficient.

### Difference in Infectivity of Sporozoites Inoculated by Mosquito Bite or Intravenous Injection

Despite our multiple publications [3], [6], [7] over the past two decades demonstrating the high level of infectivity of *P. yoelii* sporozoites when administered by intravenous (IV) injection, it was possible that the *P. yoelii* sporozoites in the mosquitoes used in our experiments (**Table 1**) were intrinsically non-infective. Therefore, in the last experiment, we dissected salivary glands from mosquitoes raised in the same container as the mosquitoes used to feed on mice (**Table 1** **, Experiment 5**), injected the sporozoites in the tail veins of mice, assessed the mice for parasitemia on days 7 and 14, and calculated the 50% infectious dose (ID_50_) of the *P. yoelii* sporozoites (**Table 4**). When 3 sporozoites were injected IV, 80% of mice developed asexual erythrocytic stage parasitemia. The ID_50_ was extremely low, and estimated to be 1.09. Therefore, the low rate of transmission of infection from mosquito bites was not due to poor infectivity of the sporozoites.

**Table 4:** Infectivity of PySPZ inoculated IV.

# of PySPZ injected intravenously into each mouse	# mice infected/# mice inoculated	% of mice with parasitemia
24	5/5	100
12	4/5	80
6	5/5	100
3	4/5	80

A sample of 15 mosquitoes was randomly taken from the same container in which the mosquitoes used to bite mice in experiment #5 were taken. Salivary glands were dissected from the 15 mosquitoes and sporozoites isolated. The indicated numbers (first column) were injected IV into mice. 7 days and 14 days later, the presence of parasitemia was determined by microscopic evaluation of thin blood smears. The ID_50_ was calculated, and determined to be 1.09 PySPZ.

## Discussion

The data reported herein indicate that the majority of *P. yoelii* sporozoites inoculated into the dermis of BALB/c mice by *A. stephensi* mosquitoes do not lead to a productive infection; they do not result in asexual erythrocytic stage infections that cause the disease known as malaria. An alternative and complimentary explanation is that a significant percentage of *P. yoelii* sporozoite-infected mosquitoes that feed do not inoculate sporozoites. Exposure to one, two or four PySPZ-infected mosquitoes led to asexual erythrocytic stage infection in 41.4% (12/29), 66.7% (22/33), and 75% (18/24) of mice respectively. Exposure to five to eight PySPZ-infected mosquitoes infected 100% (21/21) (**Table 1**). Despite the poor infectivity after one mosquito bite (41.4%), the data (**Table 4**) confirm the established fact [3], [6], [7] that almost all *P. yoelii* sporozoites in the salivary glands are infectious. When administered IV, the 50% infectious dose (ID_50_) is extremely low, and in the case of the experiment reported in **Table 4**, the ID_50_ was only 1.09 sporozoites. This is an important finding, because without such data it could be argued that only the sporozoites that are most infectious make it to the mosquito proboscis for inoculation by mosquitoes.

It was previously shown that there was a requirement for exposure to the bites of at least five *P. berghei* sporozoite-infected mosquitoes to achieve 100% infection of CD-1 mice, and fewer that three bites resulted in no infections [8]. No parallel intravenous (IV) inoculation of sporozoites was performed. However, the authors reported that in other published experiments with *P. berghei* sporozoites the ID_50_ of sporozoites inoculated intravenously to CD-1 mice was extremely high (1,700 to 11,500) indicating that the sporozoite infectivity was extremely poor [9], [10]. It should also be noted that differences in infectivity are also dependent on parasite and mouse strain, as *P. berghei* sporozoites are more infectious in H2^b^ haplotype mice such as B/6 and B/10 strains, and *P. yoelii* are more infectious in BALB/c mice. Because of the poor infectivity of *P. berghei* sporozoites, we and others [11] moved from the *P. berghei* to the *P. yoelii* rodent model system, as the high infectivity of *P. yoelii* sporozoites in mice is thought to closely resemble the infectivity of *P. falciparum* and *P. vivax* in humans and *P. knowlesi* in rhesus monkeys.

Although parallel mosquito bite and IV administration of *P. falciparum sporozoites* has not been done in recent human studies, the mosquito bite findings are consistent with published work on transmission of *P. falciparum* to volunteers by the bite of *A. stephensi*. In 1986 it was demonstrated that exposure of volunteers to five *A. stephensi* mosquitoes infected with *P. falciparum* sporozoites in their salivary glands resulted in asexual erythrocytic stage infection in all volunteers [5]. This has been repeated in more than 500 volunteers in the published literature and summarized [12], [13]. Exposure to the bites of 5 mosquitoes infected with *P. falciparum* essentially always leads to parasitemia. To determine if five mosquitoes were too many, and might overwhelm subunit vaccine-induced protective immunity, Rickman and colleagues [14] studied the capacity of 1 or 2 infected mosquitoes to transmit *P. falciparum* to volunteers. When the subjects were exposed to the bites of 1–2 *P. falciparum* (3D7 clone of the NF54 strain) infected *A. stephensi* mosquitoes, only 5 of 10 (50%) became infected with blood stage parasites. Verhage and colleagues [15] confirmed these findings. They showed that when human volunteers were exposed to the bites of 1–2 *P. falciparum* (NF54 strain) infected *A. stephensi* mosquitoes, 5 of 10 (50%) became infected and when exposed to the bites of 4–7 infected mosquitoes, 13/13 (100%) developed asexual erythrocytic stage infection. Thus, our findings in mice are analogous to what has been found in humans.

Based on work with the human malaria parasite, *P. falciparum*, in *A. freeborni*, *A. gambiae*, and *A. stephensi* mosquitoes, most malariologists have thought that mosquitoes inoculate about 5–25 sporozoites when they feed [16], [17]. Recent elegant work in mice using rodent malaria (*P. berghei*) sporozoites expressing a fluorescent protein has demonstrated that *A. stephensi* inoculate at least 100 and perhaps 300 *P. berghei* sporozoites into the dermis of mice [18]. Those findings are consistent with studies using transgenic *P. berghei* expressing β-galactosidase, and with polymerase chain reaction studies with *P. yoelii* [19], [20]. If our *P. yoelii* sporozoite-infected mosquitoes behave like the *P. berghei* and *P. yoelii*-sporozoite-infected mosquitoes reported on in these studies, and inoculate 100 sporozoites into the dermis, then our data demonstrate that in at least half the cases, none of the hundred or so sporozoites inoculated by a single mosquito invades hepatocytes, develops to a mature liver stage schizonts, and produces merozoites that infect erythrocytes. One explanation for this is that the sporozoites never migrate from the skin to the liver. It has been shown that sporozoites are deposited into the dermal tissue by *Anopheles* during probing, and slowly migrate out of the inoculation site [21], [22]. Many of these sporozoites are likely trapped in the local draining lymph nodes or encounter host defense mechanisms in the skin [23], [24], [25]. Another explanation may be that some of the failures to achieve infection were due to lack of inoculation of sporozoites rather than lack of successful migration to and development in the liver. It has been shown that approximately 22% of *P. yoelii* sporozoite-infected mosquitoes do not inoculate any sporozoites when feeding [20]. The density of salivary gland infection apparently does not impact either of these potential mechanisms, since after exposure to a single infected mosquito there was no difference in the estimated numbers of sporozoites in the glands of mosquitoes that did and did not give rise to a patent infection (Table 2).

Our goal is to develop a highly protective attenuated *P. falciparum* sporozoite vaccine. Currently the optimal dose and route of administration of *P. falciparum* sporozoites are not known. It takes exposure to greater than 950 bites from irradiated *P. falciparum* sporozoite-infected *A. stephensi* to achieve >90% protective immunity in humans [2]. Based on the analyses of efficiency of sporozoite transmission, one can estimate the dose of sporozoite vaccine required for protection. If one assumes that each bite results in sporozoite inoculation, and 5, 25, 100, or 300 sporozoites are in each inoculant, then it can be estimated that an immunizing regimen of 5,000, 25,000, 100,000 or 300,000 sporozoites would be required to achieve high-level protection in humans, if the sporozoites inoculated by needle are similar in potency to sporozoites delivered through intradermal inoculation by mosquitoes. It takes an immunizing regimen of only 3 doses of 750 *P. yoelii* sporozoites (2,250 total) administered IV to consistently achieve 90% to 100% protection in mice [3]. In contrast it takes administration of at least 1,500 attenuated *P. yoelii* sporozoites given in 4 doses (6,000 total) to mice by the ID or SC routes to achieve comparable protection (unpublished). Furthermore, at least twice as many purified, cryopreserved attenuated Py sporozoites are required for protection by ID or SC routes compared to the IV route [26]. In the long run, our goal is to achieve 90% to 100% protection of humans by administration of the fewest numbers of doses of an attenuated PfSPZ vaccine containing the lowest numbers of sporozoites. Murine and human data suggest that a significant proportion of sporozoites inoculated by mosquitoes do not lead to productive infections. Thus, as we move toward optimizing the efficiency of immunization of humans with the PfSPZ Vaccine, we are using immunity achieved by intravenous inoculation of sporozoites as the standard we would like to achieve through non-intravenous parenteral adminstration.
